# Treatment of Carbuncle and Boils

**Published:** 1890-01-25

**Authors:** 


### TREATMENT OF CARBUNCLE AND BOILS.

Some time back Prof. Vernueil, of Paris, introduced a plan of treating carbuncle by subcutaneous injections of glycerine and carbolic acid. Recently, cases cured by this method have been reported. Injections were made at several points round the inflamed region. Twenty-four hours after the first injections there were both local and general amelioration of symptoms. The formula used was glycerine and distilled water, of each fifteen grammes, crystallised carbolic acid three grammes. Although the *Journal de Medecine* mentions this treatment in a laudatory manner, we do not feel inclined to place much confidence in it. On the other hand, Ridley Dale states, in his "Epitome of Surgery," that in the early stages of a carbuncle the application of a wide zone of iodine liniment, acetum lytta, nitrate of silver, or potass c. calce, will sometimes stop its progress. Strapping in a concentric manner round the base of the carbuncle is also sometimes effective according to another surgical author. The application of arnica ointment, made of fresh arnica flowers and honey, has also been recommended. We know that small boils may sometimes be dispersed by spirits of camphor applied every three hours and allowed to dry, or by iodine paint, and it has been said that sulphide of calcium given every two or three hours generally prevents the formation of fresh boils, and reduces the area of existing boils. A better plan, however, is to prick the site of the threatened invasion with a lancet until a few drops of blood exude, which may be increased by a slight pressure. But to prove successful, this must be done as soon as possible after the first sensation of the coming furuncle is experienced. But we very much doubt if any measure has much effect in a genuine carbuncle. Surgeons, however, differ as to their use of the term, some applying it to collections which others would call merely boils. Some authors opine there is no line to be drawn between boils and carbuncles, regarding the latter as exaggeration of the former. This is correct to a certain extent; but it would appear that, while boils are sometimes connected with some debilitated condition of system, such as a malarial, a scorbutic, a syphilitic, or an anæmic state, carbuncles are generally due to other causes, such as diabetes, gout, Bright's disease, diseased meat, or they may appear as the sequel of any of the zymotic maladies. A carbuncle we regard as more especially depending on constitutional condition than boils, which are often due to inflammation of a hair follicle or a sebaceous gland. Of course, there are septic varieties of boils, which depend upon much the same causes as carbuncles. We should be glad to learn that the treatment mentioned in the *Journal de Medecine* holds its place, for at the present time surgeons are somewhat divided as to the propriety or otherwise of pursuing the old plan of making a crucial incision into a carbuncle.

